# 3,5-Dibromo-2′,3′,4′,5′,6′-penta­methyl-1,1′-biphen­yl

**DOI:** 10.1107/S1600536810015308

**Published:** 2010-05-08

**Authors:** Sorin Roşca, Marian Olaru, Ciprian I. Raţ, Cristian Silvestru

**Affiliations:** a Universitatea Babeş-Bolyai, Facultatea de Chimie şi Inginerie Chimicã, 11 Arany Janos, 400028 Cluj-Napoca, Romania

## Abstract

In the crystal structure of the title compound, C_17_H_18_Br_2_, the benzene rings are almost perpendicular [dihedral angle = 84.0 (3)°]. The crystal structure is consolidated by the presence of C—Br⋯π inter­actions.

## Related literature

For structures of related methyl substituted biphenyls, see: Fröhlich & Musso (1985 ▶); Hafelinger & Strähle (1976 ▶); Hartmann & Niemeyer (2001 ▶); Niemeyer (2006 ▶); Pickett (1936 ▶); Rathore *et al.* (1997 ▶). For background to ligands containing *m*-terphenyl groups, see: Berthiol *et al.* (2004 ▶); Cocchi *et al.* (2007 ▶); Collins *et al.* (2002 ▶); Du *et al.* (1986 ▶); Kim *et al.* (2005 ▶); Konishi *et al.* (2006 ▶); Matsumoto *et al.* (2004 ▶); Power (2004 ▶).
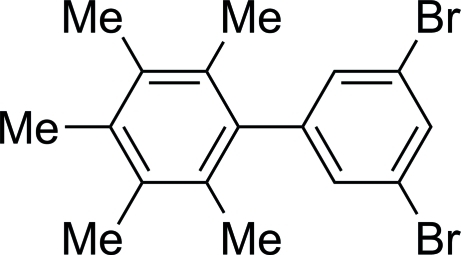


## Experimental

### 

#### Crystal data

C_17_H_18_Br_2_
*M* *_r_* = 382.13
Monoclinic, 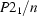

*a* = 9.011 (5) Å
*b* = 14.065 (8) Å
*c* = 12.387 (7) Å
β = 94.613 (9)°
*V* = 1564.8 (15) Å^3^
*Z* = 4
Mo *K*α radiation
μ = 5.17 mm^−1^
*T* = 297 K
0.35 × 0.32 × 0.29 mm

#### Data collection

Bruker SMART APEX CCD area-detector diffractometer
Absorption correction: multi-scan (*SADABS*; Bruker, 2000 ▶) *T*_min_ = 0.265, *T*_max_ = 0.316
10702 measured reflections
2760 independent reflections
1588 reflections with *I* > 2σ(*I*)
*R*_int_ = 0.127

#### Refinement

*R*[*F* ^2^ > 2σ(*F* ^2^)] = 0.057
*wR*(*F* ^2^) = 0.150
*S* = 0.94
2760 reflections
178 parameters
H-atom parameters constrained
Δρ_max_ = 0.67 e Å^−3^
Δρ_min_ = −0.53 e Å^−3^

### 

Data collection: *SMART* (Bruker, 2000 ▶); cell refinement: *SAINT-Plus* (Bruker, 2001 ▶); data reduction: *SAINT-Plus*; program(s) used to solve structure: *SHELXS97* (Sheldrick, 2008 ▶); program(s) used to refine structure: *SHELXL97* (Sheldrick, 2008 ▶); molecular graphics: *DIAMOND* (Brandenburg, 2009 ▶); software used to prepare material for publication: *publCIF* (Westrip, 2010 ▶) and *PLATON* (Spek, 2009 ▶).

## Supplementary Material

Crystal structure: contains datablocks I, global. DOI: 10.1107/S1600536810015308/pk2243sup1.cif

Structure factors: contains datablocks I. DOI: 10.1107/S1600536810015308/pk2243Isup2.hkl

Additional supplementary materials:  crystallographic information; 3D view; checkCIF report

## Figures and Tables

**Table 1:** C–Br⋯π inter­actions (Å, °) *Cg*2 is the centroid of the C7–C12 benzene ring.

*Y*—*X*⋯*Cg*	*Y*—*X*	*X*⋯*Cg*	*Y*⋯*Cg*	*Y*—*X*⋯*Cg*
C1—Br1⋯*Cg*2^i^	1.883 (6)	3.464 (3)	5.283 (8)	161.4 (2)

Symmetry code: (i) 1 + *x*, *y*, *z*.

**Fig. 1.**
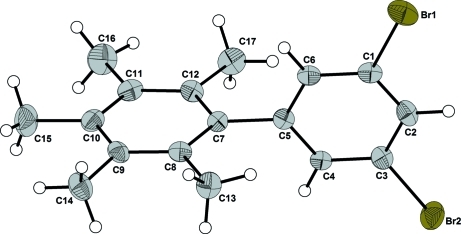
Crystal structure of the title compound with labelling and displacement ellipsoids of Br and C atoms drawn at 25 % probability level.

**Fig. 2.**
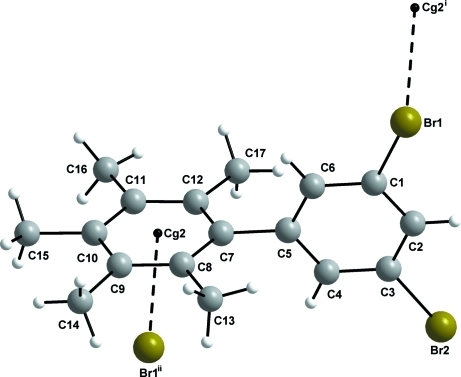
Intermolecular Br···π interactions (shown as dashed lines) in the structure of the title compound. Cg2 is the centroid of the benzene ring C7–C12. Symmetry codes: (i) 1+x, y, z; (ii) -1+x, y, z.

## supplementary crystallographic information

### Comment

Ligands containing *m*-terphenyl groups are known to stabilize many classes of compounds (Power, 2004).

The *m*-terphenyl organic precursors are prepared by coupling, via a two aryne sequence, between aromatic halides and two equivalents of Grignard reagent (Du *et al.*, 1986) or by palladium catalyzed cross-coupling reactions (Collins *et al.*, 2002; Matsumoto *et al.*, 2004; Berthiol *et al.*, 2004; Kim *et al.*, 2005; Konishi *et al.*, 2006; Cocchi *et al.*, 2007).

The title compound was obtained as a side-product in the preparation of 3,5-(2,3,4,5,6-Me_5_C_6_)_2_C_6_H_3_Br by the Suzuki cross-coupling between 2,3,4,5,6-Me_5_C_6_B(OH)_2_ and 1,3,5-Br_3_C_6_H_3_.

The dihedral angle between the planes containing the two benzene rings is 84.0 (3)° (Fig. 1), similar to those observed for the closest related compounds 2,3,4,5,6,4'-hexamethylbiphenyl tetrachloro-*p*-benzoquinone adduct (Rathore *et al.*, 1997) or 2-iodo-2',3',4',5',6'-pentamethylbiphenyl (Hartmann & Niemeyer, 2001). The bond lengths and bonding angles are normal.

In the crystal structure there are intermolecular interactions between the bromine atoms and the π electrons the methyl substituted benzene rings (Fig. 2 and Table 1).

### Experimental

Colourless crystals were obtained by slow evaporation of the solvents from solutions of the title compound in a mixture of dichloromethane and hexane. mp = 177–178 °C. ^1^H NMR (300 MHz, CDCl_3_): δ 1.95 (s, 6H), 2.25 (s, 6H), 2.30 (s, 3H), 7.24 (d, J = 1.8 Hz, 2H), 7.66 (t, J = 1.8 Hz, 1H). ^13^C NMR (75 MHz, CDCl_3_): δ 16.71, 16.98, 18.56, 123.00, 131.32, 131.58, 132.15, 132.76, 135.02, 137.14, 146.98.

### Refinement

Hydrogen atoms were placed in calculated positions with isotropic thermal parameters set at 1.2 times the carbon atoms directly attached for aromatic hydrogen atoms and 1.5 for hydrogen atoms of the methyl groups. Methyl hydrogen atoms were allowed to rotate but not to tip.

### Crystal data

**Table:**

C_17_H_18_Br_2_	*F*(000) = 760
*M**_r_* = 382.13	*D*_x_ = 1.622 Mg m^−^^3^
Monoclinic, *P*2_1_/*n*	Melting point = 451–450 K
Hall symbol: -P 2yn	Mo *K*α radiation, λ = 0.71073 Å
*a* = 9.011 (5) Å	Cell parameters from 2189 reflections
*b* = 14.065 (8) Å	θ = 2.7–22.4°
*c* = 12.387 (7) Å	µ = 5.17 mm^−^^1^
β = 94.613 (9)°	*T* = 297 K
*V* = 1564.8 (15) Å^3^	Blocks, colourless
*Z* = 4	0.35 × 0.32 × 0.29 mm

### Data collection

**Table:**

Bruker SMART APEX CCD area-detector diffractometer	2760 independent reflections
Radiation source: fine-focus sealed tube	1588 reflections with *I* > 2σ(*I*)
graphite	*R*_int_ = 0.127
φ and ω scans	θ_max_ = 25.0°, θ_min_ = 2.2°
Absorption correction: multi-scan (*SADABS*; Bruker, 2000)	*h* = −10→10
*T*_min_ = 0.265, *T*_max_ = 0.316	*k* = −16→16
10702 measured reflections	*l* = −14→14

### Refinement

**Table:**

Refinement on *F*^2^	Secondary atom site location: difference Fourier map
Least-squares matrix: full	Hydrogen site location: inferred from neighbouring sites
*R*[*F*^2^ > 2σ(*F*^2^)] = 0.057	H-atom parameters constrained
*wR*(*F*^2^) = 0.150	*w* = 1/[σ^2^(*F*_o_^2^) + (0.0001*P*)^2^] where *P* = (*F*_o_^2^ + 2*F*_c_^2^)/3
*S* = 0.94	(Δ/σ)_max_ = 0.001
2760 reflections	Δρ_max_ = 0.67 e Å^−^^3^
178 parameters	Δρ_min_ = −0.53 e Å^−^^3^
0 restraints	Extinction correction: (*SHELXL97*; Sheldrick, 2008)
Primary atom site location: structure-invariant direct methods	Extinction coefficient: 0.0150 (15)

### Special details

**Table:**

Geometry. All esds (except the esd in the dihedral angle between two l.s. planes) are estimated using the full covariance matrix. The cell esds are taken into account individually in the estimation of esds in distances, angles and torsion angles; correlations between esds in cell parameters are only used when they are defined by crystal symmetry. An approximate (isotropic) treatment of cell esds is used for estimating esds involving l.s. planes.
Refinement. Refinement of *F*^2^ against ALL reflections. The weighted *R*-factor wR and goodness of fit *S* are based on *F*^2^, conventional *R*-factors *R* are based on *F*, with *F* set to zero for negative *F*^2^. The threshold expression of *F*^2^ > σ(*F*^2^) is used only for calculating *R*-factors(gt) etc. and is not relevant to the choice of reflections for refinement. *R*-factors based on *F*^2^ are statistically about twice as large as those based on *F*, and *R*- factors based on ALL data will be even larger.

### Fractional atomic coordinates and isotropic or equivalent isotropic displacement parameters (Å^2^)

**Table:**

	*x*	*y*	*z*	*U*_iso_*/*U*_eq_	
Br1	1.08347 (7)	0.76924 (6)	0.65316 (7)	0.0714 (4)	
Br2	0.65525 (8)	1.06226 (5)	0.62304 (8)	0.0736 (4)	
C1	0.8834 (7)	0.8091 (5)	0.6463 (5)	0.0440 (16)	
C2	0.8516 (6)	0.9036 (5)	0.6407 (5)	0.0498 (17)	
H2	0.9275	0.9485	0.6415	0.06*	
C3	0.7030 (7)	0.9318 (4)	0.6337 (5)	0.0473 (16)	
C4	0.5917 (6)	0.8654 (4)	0.6332 (4)	0.0376 (15)	
H4	0.4928	0.885	0.6279	0.045*	
C5	0.6246 (6)	0.7700 (4)	0.6404 (5)	0.0392 (15)	
C6	0.7720 (6)	0.7427 (4)	0.6455 (5)	0.0428 (15)	
H6	0.7962	0.6784	0.6483	0.051*	
C7	0.5039 (6)	0.6982 (4)	0.6411 (5)	0.0394 (15)	
C8	0.4465 (6)	0.6539 (4)	0.5448 (5)	0.0445 (16)	
C9	0.3389 (6)	0.5839 (4)	0.5454 (6)	0.0468 (17)	
C10	0.2872 (7)	0.5553 (5)	0.6454 (7)	0.0544 (18)	
C11	0.3451 (7)	0.5992 (5)	0.7412 (6)	0.0484 (17)	
C12	0.4511 (6)	0.6700 (4)	0.7391 (5)	0.0444 (16)	
C13	0.5038 (7)	0.6859 (5)	0.4388 (5)	0.0609 (19)	
H13A	0.5304	0.6312	0.3981	0.091*	
H13B	0.5898	0.7255	0.4535	0.091*	
H13C	0.4276	0.7211	0.3976	0.091*	
C14	0.2779 (8)	0.5413 (5)	0.4401 (6)	0.067 (2)	
H14A	0.2468	0.5912	0.3904	0.101*	
H14B	0.1942	0.5016	0.4522	0.101*	
H14C	0.3536	0.5039	0.4101	0.101*	
C15	0.1694 (8)	0.4811 (6)	0.6491 (8)	0.086 (3)	
H15A	0.1919	0.4406	0.7106	0.129*	
H15B	0.1659	0.4437	0.584	0.129*	
H15C	0.0747	0.5111	0.6549	0.129*	
C16	0.2841 (9)	0.5705 (6)	0.8452 (7)	0.084 (3)	
H16A	0.3278	0.6092	0.9031	0.126*	
H16B	0.3073	0.5049	0.8599	0.126*	
H16C	0.178	0.5789	0.8392	0.126*	
C17	0.5056 (8)	0.7204 (5)	0.8418 (6)	0.065 (2)	
H17A	0.4264	0.7581	0.8668	0.097*	
H17B	0.5877	0.7609	0.8279	0.097*	
H17C	0.5374	0.6744	0.896	0.097*	

### Atomic displacement parameters (Å^2^)

**Table:**

	*U*^11^	*U*^22^	*U*^33^	*U*^12^	*U*^13^	*U*^23^
Br1	0.0413 (5)	0.1017 (7)	0.0722 (6)	0.0182 (4)	0.0113 (4)	−0.0009 (5)
Br2	0.0704 (6)	0.0468 (5)	0.1041 (8)	0.0015 (4)	0.0105 (5)	−0.0050 (4)
C1	0.041 (3)	0.054 (4)	0.038 (4)	0.012 (3)	0.011 (3)	−0.001 (3)
C2	0.037 (4)	0.073 (5)	0.042 (4)	−0.011 (3)	0.016 (3)	−0.005 (4)
C3	0.042 (4)	0.053 (4)	0.048 (4)	0.001 (3)	0.009 (3)	−0.005 (3)
C4	0.035 (3)	0.046 (4)	0.032 (4)	0.007 (3)	0.008 (3)	0.000 (3)
C5	0.040 (3)	0.049 (4)	0.030 (3)	0.001 (3)	0.011 (3)	0.000 (3)
C6	0.044 (4)	0.045 (4)	0.040 (4)	0.011 (3)	0.006 (3)	−0.002 (3)
C7	0.040 (3)	0.040 (3)	0.039 (4)	0.003 (3)	0.009 (3)	−0.002 (3)
C8	0.044 (4)	0.048 (4)	0.041 (4)	0.009 (3)	0.006 (3)	−0.002 (3)
C9	0.030 (3)	0.051 (4)	0.059 (5)	0.006 (3)	0.002 (3)	−0.006 (3)
C10	0.043 (4)	0.050 (4)	0.072 (6)	0.007 (3)	0.013 (4)	0.012 (4)
C11	0.042 (4)	0.056 (4)	0.050 (5)	0.015 (3)	0.022 (3)	0.008 (4)
C12	0.038 (3)	0.054 (4)	0.043 (4)	0.006 (3)	0.011 (3)	−0.004 (3)
C13	0.070 (5)	0.068 (5)	0.046 (5)	−0.005 (4)	0.016 (4)	−0.002 (4)
C14	0.061 (4)	0.071 (5)	0.068 (6)	−0.006 (4)	−0.001 (4)	−0.020 (4)
C15	0.060 (5)	0.088 (6)	0.113 (8)	−0.023 (4)	0.024 (5)	0.012 (5)
C16	0.067 (5)	0.110 (7)	0.080 (6)	−0.005 (5)	0.036 (5)	0.016 (5)
C17	0.077 (5)	0.071 (5)	0.048 (5)	0.006 (4)	0.019 (4)	−0.006 (4)

### Geometric parameters (Å, °)

**Table:**

Br1—C1	1.883 (6)	C10—C15	1.491 (9)
Br2—C3	1.887 (6)	C11—C12	1.381 (8)
C1—C2	1.361 (8)	C11—C16	1.496 (10)
C1—C6	1.370 (8)	C12—C17	1.504 (9)
C2—C3	1.392 (8)	C13—H13A	0.96
C2—H2	0.93	C13—H13B	0.96
C3—C4	1.370 (8)	C13—H13C	0.96
C4—C5	1.376 (8)	C14—H14A	0.96
C4—H4	0.93	C14—H14B	0.96
C5—C6	1.379 (7)	C14—H14C	0.96
C5—C7	1.484 (8)	C15—H15A	0.96
C6—H6	0.93	C15—H15B	0.96
C7—C12	1.397 (8)	C15—H15C	0.96
C7—C8	1.408 (8)	C16—H16A	0.96
C8—C9	1.382 (8)	C16—H16B	0.96
C8—C13	1.517 (9)	C16—H16C	0.96
C9—C10	1.417 (10)	C17—H17A	0.96
C9—C14	1.499 (9)	C17—H17B	0.96
C10—C11	1.401 (10)	C17—H17C	0.96
			
C2—C1—C6	121.0 (6)	C11—C12—C7	120.4 (6)
C2—C1—Br1	119.3 (5)	C11—C12—C17	120.2 (6)
C6—C1—Br1	119.7 (5)	C7—C12—C17	119.3 (6)
C1—C2—C3	118.6 (6)	C8—C13—H13A	109.5
C1—C2—H2	120.7	C8—C13—H13B	109.5
C3—C2—H2	120.7	H13A—C13—H13B	109.5
C4—C3—C2	120.4 (6)	C8—C13—H13C	109.5
C4—C3—Br2	120.0 (5)	H13A—C13—H13C	109.5
C2—C3—Br2	119.6 (5)	H13B—C13—H13C	109.5
C3—C4—C5	120.7 (6)	C9—C14—H14A	109.5
C3—C4—H4	119.6	C9—C14—H14B	109.5
C5—C4—H4	119.6	H14A—C14—H14B	109.5
C4—C5—C6	118.4 (6)	C9—C14—H14C	109.5
C4—C5—C7	120.6 (5)	H14A—C14—H14C	109.5
C6—C5—C7	121.0 (5)	H14B—C14—H14C	109.5
C1—C6—C5	120.9 (6)	C10—C15—H15A	109.5
C1—C6—H6	119.6	C10—C15—H15B	109.5
C5—C6—H6	119.6	H15A—C15—H15B	109.5
C12—C7—C8	118.9 (6)	C10—C15—H15C	109.5
C12—C7—C5	120.0 (6)	H15A—C15—H15C	109.5
C8—C7—C5	121.0 (6)	H15B—C15—H15C	109.5
C9—C8—C7	121.4 (6)	C11—C16—H16A	109.5
C9—C8—C13	120.2 (6)	C11—C16—H16B	109.5
C7—C8—C13	118.4 (6)	H16A—C16—H16B	109.5
C8—C9—C10	119.2 (6)	C11—C16—H16C	109.5
C8—C9—C14	119.2 (7)	H16A—C16—H16C	109.5
C10—C9—C14	121.6 (6)	H16B—C16—H16C	109.5
C11—C10—C9	119.3 (6)	C12—C17—H17A	109.5
C11—C10—C15	120.1 (7)	C12—C17—H17B	109.5
C9—C10—C15	120.7 (7)	H17A—C17—H17B	109.5
C12—C11—C10	120.9 (6)	C12—C17—H17C	109.5
C12—C11—C16	120.9 (7)	H17A—C17—H17C	109.5
C10—C11—C16	118.2 (7)	H17B—C17—H17C	109.5
			
C6—C1—C2—C3	−0.2 (9)	C7—C8—C9—C10	−1.0 (9)
Br1—C1—C2—C3	178.9 (5)	C13—C8—C9—C10	−180.0 (6)
C1—C2—C3—C4	0.4 (9)	C7—C8—C9—C14	177.9 (5)
C1—C2—C3—Br2	−178.8 (5)	C13—C8—C9—C14	−1.0 (9)
C2—C3—C4—C5	0.5 (9)	C8—C9—C10—C11	0.6 (9)
Br2—C3—C4—C5	179.7 (4)	C14—C9—C10—C11	−178.3 (6)
C3—C4—C5—C6	−1.6 (8)	C8—C9—C10—C15	179.0 (6)
C3—C4—C5—C7	179.3 (6)	C14—C9—C10—C15	0.1 (9)
C2—C1—C6—C5	−0.9 (9)	C9—C10—C11—C12	0.2 (9)
Br1—C1—C6—C5	−180.0 (4)	C15—C10—C11—C12	−178.2 (6)
C4—C5—C6—C1	1.8 (9)	C9—C10—C11—C16	177.5 (6)
C7—C5—C6—C1	−179.1 (6)	C15—C10—C11—C16	−0.9 (9)
C4—C5—C7—C12	−89.5 (7)	C10—C11—C12—C7	−0.8 (9)
C6—C5—C7—C12	91.4 (7)	C16—C11—C12—C7	−178.0 (6)
C4—C5—C7—C8	94.0 (7)	C10—C11—C12—C17	176.4 (6)
C6—C5—C7—C8	−85.0 (7)	C16—C11—C12—C17	−0.8 (9)
C12—C7—C8—C9	0.5 (9)	C8—C7—C12—C11	0.4 (9)
C5—C7—C8—C9	177.0 (5)	C5—C7—C12—C11	−176.1 (5)
C12—C7—C8—C13	179.5 (5)	C8—C7—C12—C17	−176.8 (5)
C5—C7—C8—C13	−4.0 (8)	C5—C7—C12—C17	6.7 (8)

### Table 1 C–Br···π interactions (Å, °)

*Cg*2 is the centroid of the C7–C12 benzene ring.

**Table:**

*Y*—*X*···*Cg*	*Y*—*X*	*X*···*Cg*	*Y*···*Cg*	*Y*—*X*···*Cg*
C1—Br1···Cg2^i^	1.883 (6)	3.464 (3)	5.283 (8)	161.4 (2)

Symmetry code: (i)  1+x, y, z.
